# Atlas-Assisted Bone Age Estimation from Hand–Wrist Radiographs Using Multimodal Large Language Models: A Comparative Study

**DOI:** 10.3390/diagnostics16030487

**Published:** 2026-02-05

**Authors:** Erdem Ozkan, Mustafa Koyun

**Affiliations:** Department of Radiology, Kastamonu Training and Research Hospital, Kastamonu 37150, Turkey; mustafa.koyun1@saglik.gov.tr

**Keywords:** bone age, artificial intelligence, large language model, radiology, ChatGPT, Google Gemini, Claude, Grok

## Abstract

**Background/Objectives:** Bone age assessment is critical in pediatric endocrinology and forensic medicine. Although recently developed multimodal large language models (LLMs) show potential in medical imaging, their diagnostic performance in bone age determination has not been sufficiently evaluated. This study evaluates the performance of four multimodal LLMs (ChatGPT-5, Gemini 2.5 Pro, Grok-3, and Claude 4 Sonnet) in bone age determination using the Gilsanz–Ratib (GR) atlas. **Methods:** This retrospective study included 245 pediatric patients (109 male, 136 female) under the age of 18 who underwent left wrist radiography. Each model estimated bone age using the patient’s radiograph and GR atlas as reference (atlas-assisted prompting). Bone age assessments made by an experienced radiologist using the GR atlas were evaluated as the reference standard. Performance was assessed using mean absolute error (MAE), intraclass correlation coefficient (ICC), and Bland–Altman analysis. **Results:** ChatGPT-5 demonstrated statistically superior performance, with an MAE of 1.46 years and ICC of 0.849, showing the highest alignment with the reference standard. Gemini 2.5 Pro showed moderate performance, with an MAE of 2.24 years; Grok-3 (MAE: 3.14 years) and Claude 4 Sonnet (MAE: 4.29 years) had error rates that were too high for clinical use. **Conclusions:** Significant performance differences exist among multimodal LLMs, despite atlas-supported prompting. Only ChatGPT-5 qualified as “clinically useful,” demonstrating potential as an auxiliary tool or educational support under expert supervision. Other models’ reliability remains insufficient.

## 1. Introduction

Bone age evaluation constitutes a cornerstone of pediatric clinical practice, with broad applications spanning diagnostic decision-making, treatment planning, and medicolegal assessments [1,2]. In endocrinological and metabolic disorders—including growth hormone deficiency, thyroid disease, precocious puberty, and constitutional growth delay—bone age assessment represents a critical tool in pediatric clinical evaluation. Accurate determination of skeletal maturation underpins diagnostic accuracy, enables estimation of remaining growth potential, supports prediction of final adult height, and contributes to the appropriate timing and optimization of therapeutic interventions [3,4,5]. In addition to its established role in endocrinology, bone age assessment constitutes a fundamental tool in forensic medicine, where it is routinely applied to estimate chronological age in individuals without reliable birth documentation, particularly in the context of asylum evaluations and judicial proceeding [6].

Bone age estimation is traditionally performed using radiographic analysis of the left hand and wrist based on standardized atlases, most notably the Greulich–Pyle (GP) atlas and the Gilsanz–Ratib (GR) Digital Atlas [1,2]. These atlas-based approaches assess skeletal maturation by examining ossification centers, patterns of epiphyseal development, carpal bone maturation, and growth plate closure. The GP method, first introduced in 1959, is based on visual comparison with standardized reference radiographs, whereas the more recent GR atlas provides sex-specific digital reference images at six-month intervals [1,2]. This digital framework, which stages maturity based on specific bone morphology rather than broad pattern matching, is particularly relevant when evaluating contemporary digital diagnostic tools. Alternative strategies include the Tanner–Whitehouse (TW) method, which relies on a numerical scoring system applied to individual bone [7]. Although these atlas-based techniques are widely regarded as the clinical gold standard, their application remains inherently dependent on radiologist experience and subjective interpretation, resulting in unavoidable inter- and intraobserver variability, with reported mean absolute differences ranging from 0.41 to 0.93 years even among experienced readers [8,9,10].

Driven by rapid progress in artificial intelligence (AI), automated medical image analysis has become a prominent focus in contemporary radiology and clinical research [11,12,13]. Early deep-learning approaches to automated bone age assessment primarily relied on convolutional neural networks (CNNs) trained to estimate skeletal maturity directly from hand radiographs [14,15,16]. Early fully automated deep-learning systems for bone age assessment were demonstrated in studies such as Lee et al. [14]. Landmark benchmarks, including the Radiological Society of North America (RSNA) Pediatric Bone Age Machine Learning Challenge, as well as subsequent clinical evaluations, reported mean absolute error (MAE) on the order of ~4–6 months under specific test settings [15,16]. In practice, these pipelines—largely based on CNNs—typically depend on large, carefully annotated training cohorts (often in the 10,000+ range) and task-specific model development and training workflows [15,16,17,18].

More recently, multimodal large language models (LLMs)—foundation models that can jointly process images and text—have emerged as a distinct AI paradigm with growing interest in medical application [19,20,21]. By leveraging large-scale pre-training and instruction-style prompting, foundation models can generalize in zero-shot or few-shot settings; when combined with vision–language pre-training, they can integrate image content with natural-language context in selected applications [22,23].

Recently, the utility of multimodal LLMs has been increasingly explored across a diverse spectrum of radiological tasks, ranging from the interpretation of chest radiographs to the analysis of cross-sectional imaging findings [24,25,26,27]. However, despite these promising capabilities, the diagnostic performance of such models in nuanced and clinically critical domains—such as pediatric bone age assessment—remains insufficiently characterized. In particular, a systematic benchmarking of distinct multimodal foundation models using standardized protocols that mimic real-world clinical scenarios is essential to establish the robust evidence base required for their future clinical integration.

In our study, we evaluated four state-of-the-art multimodal systems—ChatGPT-5 (OpenAI), Claude 4 (Anthropic), Gemini 2.5 Pro (Google), and Grok-3 (xAI)—all of which support image inputs together with natural-language instructions [28,29,30,31]. Unlike task-specific CNN models, instruction-tuned multimodal LLMs commonly link a pre-trained vision encoder to a language model, enabling more general-purpose vision–language inference across multiple tasks [32,33]. Crucially, the performance of these models depends on their ability to interpret natural language instructions and integrate them with visual features, offering a fundamentally different paradigm from conventional regression-based CNNs.

The aim of this investigation is to quantify the diagnostic performance of these four multimodal LLMs in bone age assessment using the GR Atlas, benchmarked against an expert radiologist reference standard.

## 2. Materials and Methods

### 2.1. Study Design and Population

**Figure 1:**
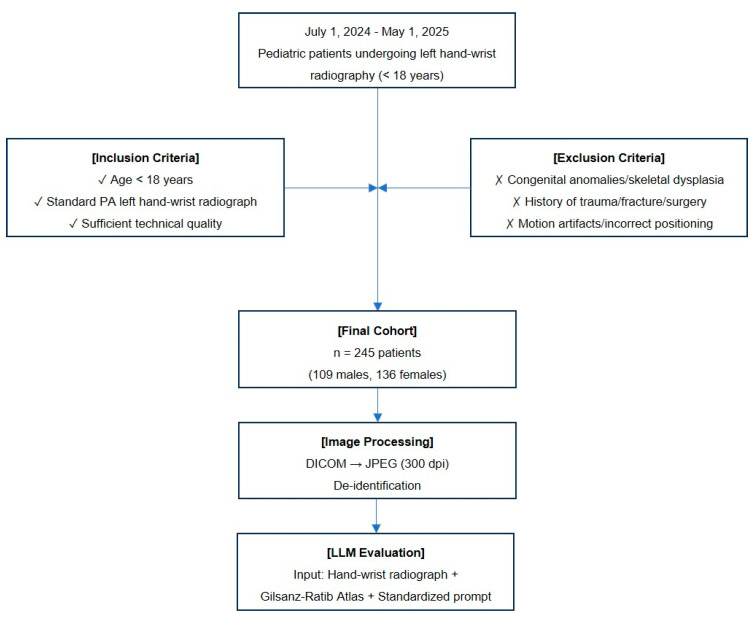
Study flowchart depicting patient selection, image processing, and LLM evaluation methodology.

This retrospective, single-center study included pediatric patients (age < 18 years) who underwent left hand–wrist radiography for bone age assessment between 1 July 2024 and 1 May 2025. A final cohort of 245 patients (109 males, 136 females) was included in the study. All examinations were performed for established clinical indications, including the evaluation of growth disorders, endocrinological–metabolic conditions, or forensic age estimation. The flowchart illustrates the systematic workflow of the study (Figure 1).

### 2.2. Inclusion and Exclusion Criteria

The inclusion criteria were defined as follows: (1) chronological age below 18 years; (2) availability of a standard posteroanterior (PA) left hand–wrist radiograph; and (3) radiographs of sufficient technical quality for diagnostic interpretation. The exclusion criteria included (1) congenital hand–wrist anatomical anomalies or skeletal dysplasias; (2) history of significant hand–wrist trauma, fracture, or prior surgical intervention; and (3) radiographs with significant motion artifacts or incorrect positioning that would preclude reliable bone age assessment.

### 2.3. Ethical Approval

The study protocol was approved by the local Institutional Review Board (Decision No.: 2025–45, Date: 18 September 2025). All procedures were conducted in full accordance with the ethical principles of the Declaration of Helsinki. Due to the retrospective nature of the study and the use of de-identified data, the requirement for informed consent was waived.

### 2.4. Radiographic Acquisition and Pre-Processing

All radiographic examinations were performed using a standardized digital radiography system (GXR82 SD; DRGEM Corp., Gwangmyeong, Republic of Korea) with acquisition parameters standardized to 40–55 kVp and 2–3 mAs. To facilitate analysis by the multimodal LLMs, the original Digital Imaging and Communications in Medicine (DICOM) images were exported from RadiAnt DICOM viewer software (version 2020.2.3, 64-bit, Medixant, Poznań, Poland) and then converted into high-resolution Joint Photographic Experts Group (JPEG) format at 300 dots per inch (dpi). Prior to evaluation, all images were rigorously de-identified to remove patient identifiers and metadata, ensuring strict data privacy and blinding the models to the patients’ chronological age.

### 2.5. Bone Age Assessment and Reference Standard Validation

Bone age of the cases was assessed by comparing the left hand–wrist radiographs with the sex-appropriate images in the GR Digital Atlas [2]. To validate the reliability of the reference standard, a quality-control subset of 30 radiographs was randomly selected for inter-observer assessment. The sample size was defined a priori in accordance with methodological guidance on ICC-based reliability study designs [34,35]. To prevent assessment bias, these radiographs were independently interpreted in a fully anonymized manner by two radiologists with 8 and 9 years of experience in musculoskeletal radiography, respectively. Both radiologists were strictly blinded to the patients’ chronological age, clinical history, previous radiological reports, and each other’s measurements. The assessments relied solely on the morphological criteria of the GR atlas. Inter-observer agreement was quantified using ICC and Pearson correlation, and further characterized by mean absolute difference and Bland–Altman analysis. Given the excellent inter-observer agreement (ICC = 0.987), bone age assessment for the entire cohort was performed by the senior radiologist (with 9 years of experience), and these measurements served as the reference standard for all subsequent LLM evaluations.

### 2.6. Large Language Models and Experimental Setup

This study benchmarked the diagnostic performance of four multimodal LLMs, selected based on their advanced vision-language processing capabilities. All models were accessed through their respective official web-based interfaces during the study period (25 September–10 October 2025).

The specific models evaluated included (1) ChatGPT-5 (OpenAI, San Francisco, CA, USA; Model version: gpt-5, accessed via chat.openai.com web interface with ChatGPT Plus subscription), (2) Google Gemini 2.5 Pro (Google LLC, Mountain View, CA, USA; Model version: gemini-2.5-pro, accessed via gemini.google.com web interface with Gemini Pro subscription), (3) Claude 4 Sonnet (Anthropic, San Francisco, CA, USA; Model version: claude-4-sonnet, accessed via claude.ai web interface with Claude Pro subscription), and (4) Grok-3 (xAI, San Francisco, CA, USA; Model version: grok-3, accessed via x.com Premium+ subscription web interface).

To ensure standardized and reproducible input across all models, a structured prompting strategy was employed. Each LLM was provided with the identical high-resolution JPEG image of the hand–wrist radiograph and the patient’s biological sex. The prompt explicitly instructed the model to analyze the image according to the standardized criteria of the GR Digital Atlas and to provide a single numerical bone age estimate. The standardized prompt used for all queries was as follows:

*“You are provided with a posteroanterior left hand and wrist radiograph of a [Male/Female] patient along with the Gilsanz–Ratib Digital Atlas as reference material. Your task: Estimate bone age by comparing the provided radiograph with the Gilsanz–Ratib Digital Atlas. Response format: ‘Estimated Bone Age: [X.X years]’. Please provide a short and concise answer using only the format above, without explanation.”*

**Figure 2:**
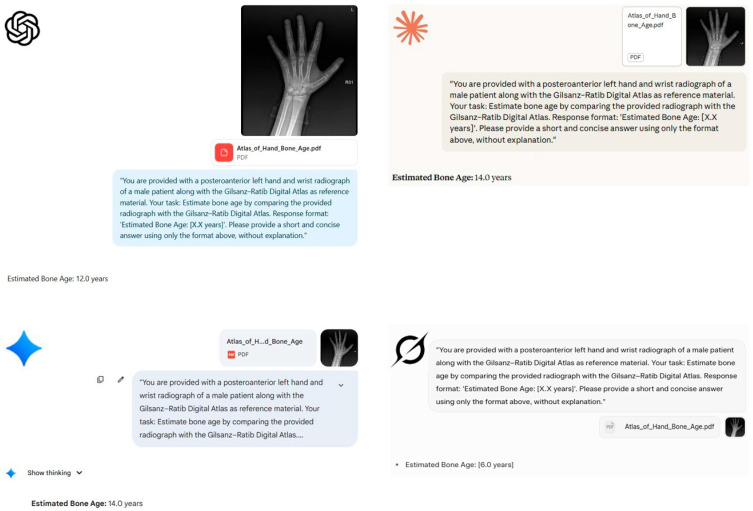
Comparison of bone age estimates made by four large language models for a 14-year-old male patient. Using the posteroanterior left hand/wrist radiograph of a male patient with a chronological age of 14 and a bone age of 14 as determined by the radiologist, bone age estimates were made by four leading large language models, using the Gilsanz–Ratib Digital Atlas as a reference. The models’ estimates are as follows: 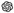 ChatGPT-5: 12.0 years, 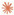 Claude 4 Sonnet: 14.0 years, 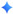 Google Gemini 2.5 Pro: 14.0 years, and 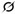 Grok-3: 6.0 years. The screenshots show each model’s response and reveal the variability in estimated bone age across platforms.

**Figure 3:**
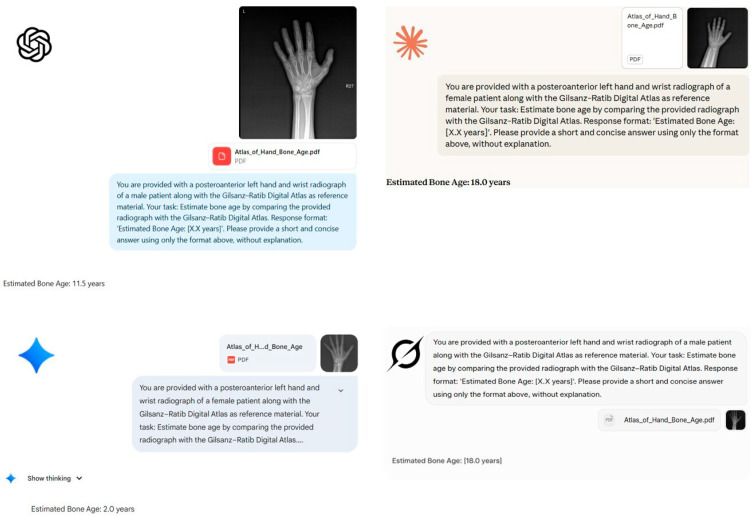
Representative examples illustrating systematic biases and random failures in bone age assessment by four large language models. Using posteroanterior left hand/wrist radiographs, bone age estimates were generated by four leading large language models and compared against the reference standard determined by an expert radiologist. The figure highlights distinct failure modes: 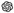 ChatGPT-5 underestimates the bone age of a 17-year-old male as 11.5 years. 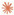 Claude 4 Sonnet significantly overestimates the bone age of an 8-year-old female as 18.0 years. 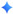 Google Gemini 2.5 Pro presents a random failure/severe underestimation, predicting 2.0 years for a 9-year-old female. 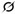 Grok-3 overestimates the bone age of a 14-year-old male as 18.0 years. These examples demonstrate the models’ significant deviations from the radiological reference standard.

A representative example of patient evaluation by the models is illustrated in Figure 2 and Figure 3.

### 2.7. Statistical Analysis

Statistical analyses were performed using IBM SPSS Statistics for Windows, version 23.0 (IBM Corp., Armonk, NY, USA). Continuous variables were expressed as mean ± standard deviation (SD) and range, while categorical variables were presented as frequencies and percentages. The normality of data distribution was assessed using the Shapiro–Wilk test.

To quantify the diagnostic accuracy of the LLMs, both the MAE and Root Mean Square Error (RMSE) were calculated. MAE represented the average absolute difference between the model’s predicted bone age and the reference standard (primary radiologist’s assessment), while RMSE was computed to penalize larger prediction errors and assess model precision. The non-parametric Friedman test was employed to compare the paired error distributions across the four LLMs. Significant differences identified by the Friedman test were further analyzed using post hoc pairwise comparisons.

Agreement between the LLM predictions and the reference standard was evaluated using the ICC (two-way mixed-effects model, absolute agreement) and the Pearson correlation coefficient (r) to assess linear relationships. Additionally, Bland–Altman analysis was conducted to visualize the agreement and quantify systematic bias.

To evaluate age and gender-specific performance variations, patients were stratified according to developmental stages defined by the Gilsanz & Ratib Digital Atlas bone age classifications. The stratification criteria were as follows: for males—Toddlers (14 months to 3 years), Pre-puberty (3–9 years), Early and Mid-puberty (9–14 years), Late puberty (14–16 years), and Post-puberty (17–19 years); for females—Toddlers (10 months to 2 years), Pre-puberty (2–7 years), Early and Mid-puberty (7–13 years), Late puberty (13–15 years), and Post-puberty (15–17 years). Mean Absolute Error (MAE) and 95% confidence intervals were calculated for each age-gender subgroup using the expert radiologist assessment as the reference standard. For subgroups with small sample sizes (*n* < 5), confidence intervals were presented as range values (minimum-maximum) to avoid statistical unreliability. A *p*-value of <0.05 was considered statistically significant for all tests.

### 2.8. Use of AI Tools

During the preparation of this manuscript, the authors used Google Gemini 2.5 Pro (Google LLC, Mountain View, CA, USA) and Claude 4 Sonnet (Anthropic, San Francisco, CA, USA) for text generation, editing, and translation. The authors have reviewed and edited all outputs and take full responsibility for the content of this publication.

## 3. Results

### 3.1. Study Population and Demographic Characteristics

A total of 245 pediatric patients were included in this retrospective study. The cohort consisted of 109 (44.5%) males and 136 (55.5%) females. The mean chronological age of the study population was 10.08 ± 3.21 years (range: 1.67–17.75 years). There was no statistically significant difference in chronological age between gender groups (*p* = 0.083).

The mean bone age determined by the reference radiologist was 10.1 ± 4.2 years. Detailed demographic characteristics are presented in Table 1.

### 3.2. Radiologist Inter-Observer Reliability

In the quality-control subset (*n* = 30), the inter-observer reliability between the two radiologists (8 and 9 years of experience) was excellent. The ICC was 0.987 (95% CI: 0.972–0.994; *p* < 0.001), indicating near-perfect consistency. The mean absolute difference between the two readers was minimal (0.43 years). Furthermore, clinical agreement (defined as a difference of ≤1.0 year) was observed in 86.7% (26/30) of the cases, confirming the robustness of the reading method. Given this high level of agreement, the primary radiologist’s measurements served as the reference standard for the entire cohort. Detailed interobserver reliability metrics are presented in Table 2.

### 3.3. Comparative Diagnostic Performance of Multimodal LLMs

The diagnostic performance metrics of the four multimodal LLMs against the reference standard are detailed in Table 3. ChatGPT-5 demonstrated statistically superior performance compared to all other models, achieving the lowest MAE of 1.46 years and the highest agreement with the reference standard (ICC = 0.849; 95% CI: 0.791–0.888; *p* < 0.001). Gemini 2.5 Pro ranked second with an MAE of 2.24 years and an ICC of 0.761 (95% CI: 0.687–0.817), showing moderate correlation compared to ChatGPT-5. This was followed by Grok-3, which exhibited significantly lower performance with an MAE of 3.14 years and an ICC of 0.379 (95% CI: 0.267–0.481), indicating poor reliability. Claude 4 Sonnet exhibited the poorest performance among all models, with the highest MAE (4.29 years) and the lowest ICC (0.216; 95% CI: 0.072–0.348), reflecting a lack of concordance with the expert radiologist. The Friedman test confirmed a statistically significant difference in error distributions across the models (χ^2^ = 150.36, *p* < 0.001), with post hoc analysis favoring ChatGPT-5. All models demonstrated statistically significant correlations with the reference standard (Pearson r ranging from 0.689 to 0.892, *p* < 0.001). However, correlation strength did not correspond with clinical agreement: despite moderate-to-strong correlations, Grok-3 (r = 0.735) and Claude 4 Sonnet (r = 0.689) exhibited poor ICC values of 0.379 and 0.216, respectively. This disparity between correlation and agreement metrics indicates that linear association does not guarantee acceptable individual-level diagnostic accuracy.

### 3.4. Age and Gender-Specific Performance Analysis of Multimodal LLMs

Stratified analysis by developmental stage revealed significant performance variations across all AI models (Table 4). ChatGPT-5 demonstrated optimal performance in pre-pubertal children of both sexes (Male: MAE 1.06, 95% CI 0.81–1.32; Female: MAE 1.25, 95% CI 0.71–1.79) and mid-pubertal females (MAE 0.97, 95% CI 0.79–1.14), consistently achieving MAE values ≤ 1.25 years. Performance degradation was observed in mid-pubertal males (MAE 1.63, 95% CI 1.31–1.96) and became pronounced in post-pubertal groups for both sexes (Male: MAE 3.73, Female: MAE 3.70). Gemini-2.5 achieved acceptable performance in male toddlers (MAE 1.00, 95% CI 0.17–1.83), late-pubertal females (MAE 0.91, 95% CI 0.35–1.47), and post-pubertal groups of both sexes (Male: MAE 2.03, 95% CI 1.25–2.82; Female: MAE 1.50, 95% CI 0.36–3.36). Claude-4 and Grok-3 demonstrated unacceptable performance across all age groups, making them unsuitable for clinical implementation. These findings suggest that ChatGPT-5 could serve as a reliable second reader for approximately 65% of the pediatric population, specifically pre-pubertal children and mid-pubertal females, while requiring expert supervision for mid-pubertal males and post-pubertal adolescents.

### 3.5. Bias and Agreement Analysis (Bland–Altman)

Bland–Altman analysis, detailed in Table 5, revealed distinct error profiles among the models. ChatGPT-5 exhibited the highest consistency with the lowest standard deviation of differences (SD = 1.85 years), showing a negligible negative bias of −0.59 years and the narrowest Limits of Agreement (LoA) range (7.25 years).

In contrast, Gemini 2.5 Pro displayed a moderate positive bias of +0.78 years, indicating a general tendency to overestimate bone age, with an intermediate LoA range (−4.51 to +6.07 years). Claude 4 Sonnet demonstrated the most substantial systematic error, consistently overestimating skeletal maturity by an average of +2.40 years with a wide error spread (SD = 4.74 years).

Notably, although Grok-3 presented a relatively low mean bias (+0.41 years), it exhibited significant instability in individual predictions. This was evidenced by a high standard deviation (SD = 4.01 years) and a remarkably wide LoA range (−7.44 to +8.26 years), suggesting that while its average error is low, its individual predictions are highly variable and unpredictable.

## 4. Discussion

This study represents one of the pioneering systematic benchmarks evaluating the diagnostic performance of four multimodal LLMs—ChatGPT-5, Gemini 2.5 Pro, Grok-3, and Claude 4 Sonnet—against an expert radiologist reference standard in pediatric bone age assessment using the Gilsanz–Ratib atlas. Our findings reveal substantial performance variation among current foundation models, with mean absolute errors ranging from 1.46 to 4.29 years.

### 4.1. Comparative Performance and the “AI Gap” in Radiology

Bone age assessment is a nuanced task requiring the synthesis of complex morphological features, traditionally associated with interobserver variability of 0.4–0.9 years even among human experts [8,9,10]. In our study, the rigorous quality-control subset demonstrated near-perfect agreement between radiologists (ICC = 0.987; MAE = 0.43 years), establishing a robust ground truth.

In stark contrast, a significant performance gap remains between this human “gold standard” and generalist AI. Even the top-performing model, ChatGPT-5, yielded an MAE of 1.46 years (ICC: 0.849)—three to five times higher than specialized CNNs. This error margin is substantially wider than that of task-specific CNNs, which consistently achieve MAEs in the range of 4 to 6 months (0.3–0.5 years) in landmark benchmarks such as the RSNA Pediatric Bone Age Challenge [15,16]. The performance disparities among the tested LLMs were even more striking: Gemini 2.5 Pro (MAE: 2.24 years, ICC: 0.761), Grok-3 (MAE: 3.14 years, ICC: 0.379), and Claude 4 Sonnet (MAE: 4.29 years, ICC: 0.216) demonstrated progressively deteriorating accuracy. This discrepancy highlights a fundamental distinction in AI architectures: while specialized CNNs are trained via supervised learning on massive, annotated datasets for pixel-level regression, multimodal LLMs operate as generalist “reasoning engines.” These results suggest that, despite advanced vision encoders, many general-purpose LLMs currently lack the fine-grained feature discrimination required to match the precision of dedicated narrow AI models in specialized radiological tasks [19,20]. The marked performance disparities among the four LLMs (MAE ranging from 1.46 to 4.29 years) merit closer examination of the underlying architectural factors. The superior performance of ChatGPT-5 can be attributed to its advanced architectural integration of visual and textual modalities. Unlike earlier models that often process visual inputs as separate tokens, ChatGPT-5 likely utilizes enhanced cross-modal alignment mechanisms optimized for complex reasoning tasks [36]. This allows the model to better synthesize the semantic descriptions in the prompt with the fine-grained radiological features in the image, effectively mimicking the ‘feature extraction’ process of a radiologist. In contrast, models like Claude 4 Sonnet appeared to struggle with this multimodal grounding, leading to hallucinations where the generated text description did not match the actual visual evidence.

### 4.2. Methodology: The Impact of Atlas-Assisted Prompting

A distinguishing methodological feature of this study was the “atlas-assisted” evaluation protocol. Unlike standard zero-shot benchmarks where models rely solely on pre-trained weights, we simulated a comparative radiological workflow by providing the LLMs with both the patient radiograph and the corresponding GR digital reference plates.

Recently, Büyüktoka and Salbaş evaluated the zero-shot performance of multimodal LLMs, including Gemini 2.5 Pro and ChatGPT-4.5, on the RSNA pediatric dataset [37]. In their analysis, even the top-performing model (Gemini 2.5 Pro) exhibited a high mean absolute error of approximately 2.37 years, leading them to conclude that current models are unsuitable for clinical use. In contrast, our study achieved a significantly lower MAE of 1.46 years with ChatGPT-5. This substantial performance gain in our cohort may be attributed to two key factors: the superior reasoning capabilities of the newer ChatGPT-5 architecture and, crucially, our implementation of an “atlas-assisted” prompting protocol, which provides the model with a reference standard rather than relying solely on internal weights.

The superior performance of ChatGPT-5 and, to a lesser extent, Gemini 2.5 Pro, suggests that these models possess a more advanced capability to integrate multimodal inputs [27,32]—performing visual cross-referencing between the “patient” and “atlas” images. However, this scaffolding proved insufficient for other models. Claude 4 Sonnet exhibited a massive systematic failure with an MAE of 4.29 years and a substantial positive bias (+2.40 years). Such systematic overestimation is clinically dangerous, as it could lead to the misdiagnosis of pathologies like precocious puberty or result in inappropriate aggressive treatments [3,4]. Similarly, while Grok-3 showed a low mean bias (+0.41 years), its high standard deviation and wide LoA (15.70 years) indicate a stochastic, unpredictable output pattern, rendering it unreliable despite the atlas support. This underscores that access to reference material cannot compensate for a model’s intrinsic limitations in radiological pattern recognition.

### 4.3. Age-Dependent Variability and Clinical Implications

Consistent with previous literature on deep learning in auxology, peri-pubertal periods (approx. 10–15 years) present unique challenges for automated bone age assessment due to subtle, rapid morphological changes in carpal bones and epiphyses [5,14,16]. This developmental phase involves transitional stages that mirror the “fine-grained” vs. “coarse-grained” visual recognition challenge seen in computer vision research [14,17]. However, our stratified analysis revealed that performance degradation during this critical period was not uniform across all models or gender groups.

Our detailed age and gender analysis demonstrated distinct patterns of model reliability. ChatGPT-5 maintained excellent accuracy across multiple subgroups, including pre-pubertal children of both sexes (Male: MAE 1.06 years; Female: MAE 1.25 years) and mid-pubertal females (MAE 0.97 years), while performance declined in mid-pubertal males (MAE 1.63 years), suggesting gender-specific developmental timing affects model interpretation. Notably, Gemini-2.5 achieved its best overall performance in late-pubertal females (MAE 0.91 years), contradicting the assumption of peri-pubertal degradation. The most pronounced difficulties occurred in post-pubertal groups where epiphyseal fusion creates interpretive challenges that exceed current model capabilities.

These findings reveal clear differences in the potential clinical application of the models. ChatGPT-5 demonstrated the highest level of proficiency among the tested LLMs, showing significant promise as a ‘second reader’ or educational adjunct [13], particularly for pre-pubertal children of both sexes and mid-pubertal females. These subgroups represent approximately 65% of the pediatric population, where the model achieved MAE values comparable to the lower bounds of human variability. However, users must be cognizant of its slight tendency to underestimate age (bias: −0.59 years). Crucially, expert supervision remains mandatory, especially for mid-pubertal males and post-pubertal adolescents, where performance significantly deteriorates due to complex ossification patterns. Gemini 2.5 Pro exhibited intermediate utility, achieving acceptable accuracy in selective subgroups such as male toddlers, late-pubertal females, and post-pubertal patients, yet it lacks the consistency required for broad implementation without mandatory expert revision. Conversely, Grok-3 and Claude 4 Sonnet failed to meet the minimum accuracy requirements for even rough screening purposes [6] across all age and gender subgroups. Consequently, these two models are currently deemed unsuitable for clinical tasks in pediatric bone age assessment.

Beyond the technical limitations of LLMs, the biological variability of skeletal maturation across different populations remains a cornerstone of bone age assessment research. Western clinical literature has extensively documented that ‘biological age’ is significantly modulated by ethnic, geographic, and socioeconomic factors [9,38]. For instance, longitudinal studies in North American and European cohorts have shown that children of African and Hispanic descent often exhibit accelerated skeletal maturation compared to their Caucasian counterparts, upon whom the GR and Greulich–Pyle standards were originally based [38]. Furthermore, research emphasizes that environmental factors, including nutrition and the timing of the pubertal growth spurt, vary geographically, potentially leading to systemic deviations when a single-population atlas is applied globally [2]. These established variations suggest that the ‘standardized’ reference points of the GR atlas may not perfectly align with the developmental trajectory of our Turkish cohort. Consequently, the performance of LLMs in this study must be interpreted within the context of this potential geographic-biological bias, highlighting the necessity for future AI models to be trained on more diverse, multi-ethnic datasets to ensure global diagnostic reliability.

### 4.4. Strengths and Limitations

The primary strength of this investigation lies in its comprehensive comparative design. To the best of our knowledge, this is the first study to benchmark the diagnostic performance of these four distinct multimodal foundation models (ChatGPT-5, Gemini 2.5 Pro, Claude 4 Sonnet, and Grok-3) simultaneously for pediatric bone age assessment. By including newer and less-evaluated models such as Grok-3 and Claude 4 Sonnet, our study addresses a critical gap in the current radiological AI literature, which has predominantly focused on OpenAI and Google ecosystems. Additionally, the adoption of an “atlas-assisted” prompting protocol mimics real-world radiological practice, providing a more ecologically valid assessment of LLM capabilities compared to standard zero-shot benchmarks.

However, this study is subject to several limitations. First, the retrospective, single-center design may limit the generalizability of findings to diverse ethnic populations, as skeletal maturation norms can vary globally [9]. Second, we utilized commercial, closed-source models; thus, the exact composition of their training data remains opaque. It is possible that the Gilsanz–Ratib atlas images were present in the models’ pre-training datasets. However, since the atlas images were explicitly provided within each prompt and the patient radiographs were strictly unseen data from 2024 to 2025, the task fundamentally required visual comparison and reasoning capabilities rather than atlas memorization. Moreover, the marked performance disparity among models receiving identical inputs (e.g., ChatGPT-5 vs. Claude 4 Sonnet) suggests that multimodal reasoning architecture, rather than simple data recall, is the primary determinant of success. Third, due to the retrospective design and anonymization protocols, clinical diagnoses were not correlated with bone age findings. Therefore, this study focused strictly on the agreement between AI models and the radiologist’s assessment, rather than the accuracy of age prediction relative to chronological age. Future research should explore “Chain-of-Thought” prompting, where LLMs are instructed to explicitly identify and describe specific ossification centers before generating an age estimate, which could potentially improve interpretability and accuracy [22,38]. Fourth, although our quality-control subset demonstrated excellent inter-observer agreement (ICC = 0.987), establishing the reference standard based primarily on a single expert radiologist’s assessment for the full cohort represents a limitation. However, the sample size for this reliability subset (*n* = 30) was determined a priori based on statistical power calculations for ICC reliability studies [34,35], ensuring that this subset was sufficient to validate the primary reader’s consistency. Finally, the reliance on the Gilsanz–Ratib atlas, which was standardized on a North American Caucasian population, represents a significant geographic limitation. Given the established influence of ethnicity and socioeconomic factors on bone age [38], the reference standards used may not fully capture the specific maturation patterns of Turkish children, potentially introducing an inherent biological bias into our accuracy metrics.

## 5. Conclusions

In conclusion, this study demonstrates that while multimodal LLMs such as ChatGPT-5 show emerging promise in pediatric bone age assessment, they have not yet achieved the diagnostic precision of experienced radiologists or specialized CNNs. The significant performance disparities—ranging from the potentially useful (ChatGPT-5) to the clinically unreliable (Claude 4 Sonnet)—highlight that “multimodal” capability is not uniform across all foundation models. While the “atlas-assisted” approach validates the potential of LLMs to perform comparative reasoning, significant architectural improvements in fine-grained visual processing are necessary before these tools can be safely integrated into autonomous diagnostic workflows. Nevertheless, given the rapid evolution of multimodal LLM technology and ongoing model optimizations, these models hold significant potential to evolve into reliable adjunctive tools for bone age assessment in the near future.

## Figures and Tables

**Table 1:** Demographic characteristics of the study population.

Characteristic	Male (*n* = 109)	Female (*n* = 136)	Total (*n* = 245)	*p*-Value
**Chronological age (years)**				
Mean ± SD	10.49 ± 3.94	9.74 ± 2.43	10.08 ± 3.21	0.083 *
Range (min–max)	1.67–17.75	3.67–15.83	1.67–17.75	-
**Bone age (radiologist reference) (years)**				
Mean ± SD	10.2 ± 4.1	10.0 ± 4.3	10.1 ± 4.2	0.655 *

SD: standard deviation; * calculated using independent samples *t*-test.

**Table 2:** Interobserver reliability metrics and descriptive statistics for radiologist assessments.

Parameter	Value
**Quality-control subset (** ***n*** **= 30)**	
Radiologist 1 (R1), bone age (years), Mean ± SD	9.9 ± 4.1
Radiologist 2 (R2), bone age (years), Mean ± SD	9.8 ± 4.0
**Interobserver agreement (** ***n*** **= 30)**	
Intraclass correlation coefficient (ICC)	0.987 (95% CI: 0.972–0.994)
Mean absolute difference	0.43 years
Bland–Altman bias (R1–R2)	0.03 years
95% limits of agreement	−1.48 to +1.55 years
**Full cohort (** ***n*** **= 245)**	
Primary radiologist, bone age (years), Mean ± SD	10.2 ± 3.9 years
Range	1.5–18.0 years

SD: standard deviation; CI: confidence interval; ICC: intraclass correlation coefficient.

**Table 3:** Comparative diagnostic performance metrics of multimodal large language models against the expert radiologist reference standard: overall cohort analysis.

Models	MAE (years)	RMSE (years)	ICC (95% CI) *	Pearson r	*p*-Value **
ChatGPT-5	1.46 ± 1.28	1.94	0.849 (0.791–0.888)	0.892	<0.001
Gemini 2.5 Pro	2.24 ± 1.69	2.81	0.761 (0.687–0.817)	0.784	<0.001
Grok-3	3.14 ± 2.51	3.45	0.379 (0.267–0.481)	0.735	<0.001
Claude 4 Sonnet	4.29 ± 3.12	4.32	0.216 (0.072–0.348)	0.689	<0.001

MAE: mean absolute error; RMSE: root mean square error; ICC: intraclass correlation coefficient (single measures, absolute agreement); CI: confidence interval; * ICC values indicate agreement with the reference radiologist; ** significance level for the correlation.

**Table 4:** Mean Absolute Error (MAE) and 95% Confidence Intervals stratified by sex and developmental stage.

Sex	Age Group	Age Range	*n*	ChatGPT-5 MAE (Years) (95% CI)	Claude-4 MAE (Years) (95% CI)	Gemini-2.5 MAE (Years) (95% CI)	Grok-3 MAE (Years) (95% CI)
**Male**	Toddlers	14 mo–3 yr	10	1.80 (1.21–2.39)	9.40 (6.53–12.27)	**1.00 (0.17–1.83)**	4.80 (3.76–5.84)
	Pre-puberty	3–9 yr	39	**1.06 (0.81–1.32)**	6.42 (5.34–7.51)	2.10 (1.57–2.63)	3.67 (2.90–4.43)
	Early and Mid-puberty	9–14 yr	45	**1.63 (1.31–1.96)**	2.34 (1.81–2.88)	2.41 (1.89–2.93)	2.98 (2.25–3.70)
	Late-puberty	14–16 yr	-	-	-	-	-
	Post-puberty	17–19 yr	15	3.73 (2.92–4.54)	2.40 (1.59–3.21)	**2.03 (1.25–2.82)**	6.17 (4.43–7.90)
**Female**	Toddlers	10 mo–2 yr	2	0.50 (0.00–1.00) *	7.25 (4.00–10.50) *	0.75 (0.50–1.00) *	7.00 (5.00–9.00) *
	Pre-puberty	2–7 yr	14	**1.25 (0.71–1.79)**	4.89 (2.51–7.27)	2.29 (1.37–3.20)	4.18 (2.43–5.93)
	Early and Mid-puberty	7–13 yr	104	**0.97 (0.79–1.14)**	3.91 (3.45–4.37)	2.56 (2.22–2.90)	2.02 (1.73–2.31)
	Late-puberty	13–15 yr	11	2.77 (1.73–3.82)	4.23 (2.39–6.06)	**0.91 (0.35–1.47)**	3.00 (1.92–4.08)
	Post-puberty	15–17 yr	5	3.70 (2.34–5.06)	5.60 (2.22–8.98)	**1.50 (−0.36–3.36)**	7.10 (0.82–13.38)
**Total**	All Groups	10 mo–19 yr	245	**1.46 (1.30–1.62)**	4.29 (3.90–4.68)	2.24 (2.03–2.45)	3.14 (2.82–3.45)

MAE: mean absolute error; CI: confidence interval; mo: months; yr: years, *n*: number. Note: Values are presented as Mean Absolute Error (95% Confidence Interval) in years. Developmental stages and age ranges are defined according to the Gilsanz & Ratib Bone Age Atlas. Bold values indicate the model with the lowest MAE (best performance) for the specific subgroup. * For subgroups with a small sample size (*n* < 5), values are presented as Mean (Minimum–Maximum) instead of 95% CI to avoid statistical unreliability.

**Table 5:** Bland–Altman analysis results: systematic bias and limits of agreement (LoA) for the entire study cohort.

Models	Mean Bias (Years) *	SD of Bias	95% Limits of Agreement (LoA)	LoA Range (Width)
ChatGPT-5	−0.59	1.85	−4.21 to +3.04	7.25 years
Gemini 2.5 Pro	+0.78	2.70	−4.51 to +6.07	10.58 years
Grok-3	+0.41	4.01	−7.44 to +8.26	15.70 years
Claude 4 Sonnet	+2.40	4.74	−6.89 to +11.69	18.58 years

*: negative bias indicates underestimation; positive bias indicates overestimation of bone age relative to the radiologist. SD: standard deviation.

## Data Availability

The data presented in this study are available on request from the corresponding author. The data are not publicly available due to privacy.
